# Draft Genome Sequence of *Ochrobactrum* sp. Strain MC-1LL, a Bacterial Strain with Antimicrobial Properties, Isolated from Marine Sediments in Nigeria

**DOI:** 10.1128/MRA.00425-20

**Published:** 2020-05-28

**Authors:** Bamidele Tolulope Odumosu, Uzoigwe Ngozika Augusta, Carolina Cano-Prieto, Anina Buchmann, Kay Nieselt, Harald Gross

**Affiliations:** a Department of Microbiology, University of Lagos, Akoka-Yaba, Lagos State, Nigeria; b Department of Pharmaceutical Biology, Pharmaceutical Institute, University of Tübingen, Tübingen, Germany; c Integrative Transcriptomics, Institute for Bioinformatics and Medical Informatics, University of Tübingen, Tübingen, Germany; Georgia Institute of Technology

## Abstract

Here, we report a 4.3-Mb draft genome sequence of a potential new *Ochrobactrum* species, which clarified its taxonomic position and gave insight into the complete secondary metabolite production capacity of the strain.

## ANNOUNCEMENT

As part of our ongoing efforts to investigate natural products from underexplored bacteria with significant applications in the pharmaceutical-medical or agricultural context (1–4), we recently isolated and characterized samples from Lagos Lagoon, Nigeria (5). In a subsequent screening panel (5), the isolate MC-1LL exhibited antibacterial activity. Initial taxonomic classification efforts, based on 16S rRNA, gave inconclusive results. BLAST comparison of the obtained 16S rRNA sequence showed that the strains most closely related to MC-1LL are *Ochrobactrum* sp. strain CGL-X (100.00% identity, DQ305290), *Ochrobactrum* sp. strain LJ-C (99.79% identity, MF062571), and *Brucella* sp. strain YBJCA-1 (99.93% identity, DQ305284). However, Ochrobactrum oryzae MTCC 4195 (NR_042417) shows only 97.68% 16S rRNA sequence identity to strain MC-11LL but represents the most closely related validly described type strain. *Ochrobactrum* and *Brucella* species both represent taxonomically closely related Gram-negative pathogens which can be difficult to discriminate (6, 7) and which are chemically underexplored. Therefore, we aimed to determine the whole-genome sequence of this strain to clarify its taxonomic position, to reveal the genetic background of its antibacterial properties, and to shed more light on the complete biosynthetic capacity for secondary metabolism.

The strain was isolated from the subtidal zone of the Lagos Lagoon front toward the Ebute Metta axis of the marine at a depth of 50 m below the surface. One gram of marine sediments was collected in a ziplock bag and maintained on ice until it was transported to the laboratory, where the sample was processed immediately. A total of a 0.1-ml aliquot obtained from a 10^6^ serial dilution of the marine sediments in tryptic soy broth was spread onto marine agar (Oxoid, UK) and incubated at 30°C for 21 days, and distinguishing colonies and morphological appearances started appearing after 7 days of incubation. Strain MC-1LL was isolated as a single colony and repeatedly subcultured on the same medium to check and confirm its purity.

Genomic DNA of strain MC-1LL was harvested from an overnight culture grown at 37°C in 15 ml lysogeny broth (8) on a rotary shaker (220 rpm) and isolated as previously described (4). The sequencing library was prepared using the Pacific Biosciences protocol for preparing multiplexed microbial SMRTbell libraries, barcoded hairpin adapters, and a PacBio barcoded adapter. The 6-kb multiplex library was sequenced on a Pacific Biosciences Sequel instrument using v3.0 chemistry, including Sequel polymerase v3.0 and one single-molecule real-time (SMRT) cell v3, resulting in 500,593 reads with a median read length of 4,542 bp. No quality filtering was conducted; however, subreads shorter than 50 bp were discarded. The remaining PacBio long reads were assembled using SMRTLink v7.0.1 and the Hierarchical Genome Assembly Process v4.0 (HGAP4.0) with default parameters and an expected 5-Mbp genome size based on previously determined *Ochrobactrum* and *Brucella* genome sizes, which range commonly from 3.2 to 5 Mbp (9–12). Overall, the reads were assembled into a 4,329,544-nucleotide draft genome with 489-fold coverage. The resulting sequence of strain MC-1LL consists of 5 contigs with a G+C content of 57.8%. Gene functional annotation using the NCBI Prokaryotic Genome Annotation Pipeline (PGAP) v4.11 (13, 14) identified 4,026 coding genes.

An automated genome-based taxonomic analysis of strain MC-1LL, employing the Type Strain Genome Server (TYGS) (15), revealed that Ochrobactrum intermedium LMG 3301 represents the closest related type strain of strain MC-1LL. In pairwise comparisons, independent of the applied Genome BLAST Distance Phylogeny (GBDP) formula, the digital DNA-DNA hybridization (dDDH) values *d*_0_, *d*_4_, and *d*_6_ did not exceed 66.9%. Since these values are below the species threshold of 70%, strain MC-1LL represents a candidate new *Ochrobactrum* species. This finding was complemented by an analysis of the average nucleotide identity (ANI) using autoMLST (16), which revealed that the MC-1LL genome sequence had 91.7% ANI to *O. intermedium* LMG 3301^T^ and 88.3% ANI to Ochrobactrum anthropi ATCC 49188^T^. Both ANI values are well below the boundary of 95 to 96% ANI for species delineation, thereby corroborating the TYGS results. Since the genomes of *Ochrobactrum* spp. have been previously described as complex with two independent circular chromosomes (17), we hypothesize that the five assembled contigs most likely represent parts of two chromosomes.

Automated secondary metabolism analysis using antiSMASH 5.0.0 (18) with default settings predicted four biosynthetic gene clusters encoding a beta-lactone, a terpene, an arylpolyene (19), and an acyl amino acid-based compound.

### Data availability.

This whole-genome sequencing (WGS) project has been deposited at DDBJ/ENA/GenBank under the accession number JAAVWX000000000. The raw sequencing data are available from the Sequence Read Archive (SRA) under the accession number SRR11514879.
